# The Schistosomiasis Consortium for Operational Research and Evaluation 2008–2020: Approaches, Experiences, Lessons, and Recommendations

**DOI:** 10.4269/ajtmh.19-0786

**Published:** 2020-05-12

**Authors:** Sue Binder, Carl H. Campbell, Tamara S. Andros, Jennifer D. Castleman, Nupur Kittur, Charles H. King, Daniel G. Colley

**Affiliations:** 1 Schistosomiasis Consortium for Operational Research and Evaluation, Center for Tropical and Emerging Global Diseases, University of Georgia, Athens, Georgia;; 2 Center for Global Health and Diseases, Case Western Reserve University School of Medicine, Cleveland, Ohio;; 3 Department of Microbiology, University of Georgia, Athens, Georgia

## Abstract

For the past 10 years, the Schistosomiasis Consortium for Operational Research and Evaluation (SCORE), funded by the Bill & Melinda Gates Foundation, has been supporting operational research to provide a stronger evidence base for controlling and moving toward elimination of schistosomiasis. The SCORE portfolio was developed and implemented with engagement from many stakeholders and sectors. Particular efforts were made to include endemic country neglected tropical disease program managers. Examples of the challenges we encountered include the need to balance rigor (e.g., conducting large cluster-randomized trials) with ensuring relevance to real-world settings, allowing for local contexts while standardizing key study aspects, adjusting to evolving technologies, and incorporating changing technologies into multiyear studies. The Schistosomiasis Consortium for Operational Research and Evaluation’s findings and data and the collected specimens will continue to be useful in the years to come. Our experiences and lessons learned can benefit both program managers and researchers conducting similar work in the future.

## INTRODUCTION

The Schistosomiasis Consortium for Operational Research and Evaluation (SCORE) was funded in December 2008 by the Bill & Melinda Gates Foundation (BMGF) to support research that would provide evidence leading to improved means to reduce morbidity from schistosomiasis and to move toward elimination, with a focus on countries in Africa.^1^ The SCORE has been managed by a small secretariat, headquartered at the University of Georgia (UGA). This article discusses our experiences in carrying out the complex portfolio of research activities and the secretariat’s recommendations that result from these experiences. Some of these, and the associated benefits and considerations, are listed in Table 1 and described in the following text.

**Table 1:** Recommendations arising from the experiences of the SCORE secretariat and examples of these experiences

Recommendation	Benefits and considerations	Examples of SCORE experiences
**Broad engagement**		
Engage the broad community —both those who are or will be funded and those who will not	Broad input helps ensure that the most relevant questions are asked. It supports dissemination and is more likely to impact practice and follow-up research.	Broad engagement took time and resources. The BMGF grant encouraged rapid roll-out of studies, requiring the SCORE secretariat to balance the time required to get broad input with the need to quickly implement sub-awards.
		Involvement of the WHO in planning meetings and as an ad hoc advisory committee member stimulated development of guidance related to point-of-care circulating cathodic antigen. Results from the large field studies are currently being used to inform new guidelines.
Partner with country NTD control programs in the research and to explore the implications of findings	Engaging NTD control programs ensures relevance of the studies and helps speed uptake of results. Because NTD control programs are critical to the WHO’s efforts, their involvement provides additional links to WHO recommendation and guideline development.	Engaging NTD control program managers who had not previously been involved with research in their countries required time and resources. In some places, SCORE researchers had difficulty engaging program managers or engagement was limited because of frequent program manager turnover.
**Trade-offs and balances**		
Recognize the limitations imposed by resources and capacity, and design studies accordingly	An overly ambitious study is likely to fail.	The SCORE gaining and sustaining control studies were considered to be at the limit of what the research sites could handle. Some critical questions had to be excluded. All the studies were completed and provided useful data.
Balance rigor and relevance	The large field studies all used a cluster-randomized design—which is considered a strong research design. The main intervention, MDA, was implemented in the context of ongoing programs.	The randomization did not always result in balanced confounders among arms. Unexpectedly, high variability in village responses to MDA resulted in less power than anticipated. Integration with country programs took additional time and resources.
Weigh the risks and potential knowledge gained by conducting field studies in places that might have “red flags” on first consideration	Initially, SCORE and others were reluctant to conduct the major elimination study in Zanzibar. Some of the sites with little infrastructure or with instability (e.g., Mozambique) appeared to offer tremendous opportunities for the gaining and sustaining control studies.	The concern that work in Zanzibar would not be seen as generalizable appears now to be unfounded; the long history of MDA and political commitment helped make the study successful. In Mozambique, lack of school attendance and inadequate sensitization led to very low coverage levels. Nevertheless, the impact of MDA was clear. Political instability led to a delayed start of the sustaining control study in Côte d’Ivoire.
In multiyear studies, be visionary but cautious in incorporating new technologies	Researchers and staff appreciate the opportunity to be on the cutting edge of using new technologies. Significant resources can be saved if time allows delaying efforts to take advantage of rapidly developing technologies.	Had personal digital assistants been used for data collection in the gaining and sustaining studies, they would have been obsolete by the time the studies ended. The gaining and sustaining control studies were being implemented as smartphone data collection systems were developing. Keeping the systems simple would have made development and testing easier. For population genetics studies, it was possible to wait for technology to catch up to the study needs, resulting in more efficient processing of specimens, but creating a delay in obtaining results.
**Management and oversight**		
Include adequate resources and personnel centrally for early and frequent on-site visits, especially for large field studies	Limiting secretariat visits and staff for close oversight allows for more resources for study implementation and possibly for more ownership of the studies by sites.	Greater oversight would have led to earlier identification of problems and higher quality implementation. Principal investigators and northern partner institutions were not always as attentive to the protocol and quality of study implementation as expected, and greater oversight might have addressed this issue.
Ensure resources are available for contingencies and opportunities	Resources are needed to address perturbations, for example, study delays due to political and other concerns, and to use the research infrastructure to address questions that arise in the course of the research.	Without the flexibility on the part of the BMGF and SCORE to address unexpected issues, some studies would not have been completed. Some critical SCORE results have come from studies added as opportunities or new questions were identified during the course of the core studies.
**Intervention implementation and measurement**		
Ensure the primary intervention is adequately resourced and has appropriate oversight	SCORE attempted to walk a fine line between encouraging research-level implementation of interventions and implementation that might be realistic for programs.	In some of the gaining and sustaining control studies, poor coverage may have contributed to failure to see differences in outcomes among study arms. More resources and earlier implementation of snail control and behavioral interventions in the Zanzibar elimination study might have resulted in a greater impact in the arms involving these interventions.
Invest in measuring MDA quality and process measures related to MDA coverage	Better process measures related to the steps required for a successful MDA (e.g., what is being done for sensitization) and better coverage data might help identify problems earlier in the studies and provide information that could help explain results.	Collection of quality data requires significant resources. In places like Mozambique, with frequent large population shifts, an attempt to estimate denominators without investing in a repeat census was not successful. Coverage estimates by community drug distributors were shown to be overestimates in the Kenya gaining control study. Formal coverage survey evaluators should be considered for studies in which MDA is a primary intervention.
**Measurement and analysis of outcomes**		
Define data elements in advance and set quality standards	Up-front investment in study design ensures higher quality and reduces time spent cleaning data and resolving data issues.	Initially, SCORE relied on sites with long histories of collecting data on similar outcomes, for example, infection status, to provide quality data. This did not work adequately, requiring significant amounts of time reconstructing and fixing issues related to data quality.
Develop SAPs before analysis of field study data	Explicitly defined analyses are standard for clinical trial research to avoid selective publication of only those results that support the study’s hypotheses. Analyses and publications using the SAP allows for easy comparisons of results from different studies using the same design.	Although the SCORE studies are somewhat structured like clinical trials, many aspects of the studies are not controlled. For the SCORE studies, some investigators questioned the time and effort involved in developing the SAPs. However, the SAPs ultimately were useful in structuring analysis and allowing for comparisons among studies.
Ensure that data instruments and collection for additional analyses are adequate for the intended purpose and provide training needed to collect quality data	The SCORE field studies provided opportunities to evaluate questions such as the cost of alternative approaches to MDA and whether village-level indicators could help in assessing force of transmission.	The data collection systems for cost and village-level indicators in the gaining and sustaining studies were used in most sites without adequate pretesting.

BMGF = Bill & Melinda Gates Foundation; MDA = mass drug administration; NTD = neglected tropical diseases; SAPs = standardized analysis plans; SCORE = Schistosomiasis Consortium for Operational Research and Evaluation; WHO = World Health Organization.

## THE SCORE PORTFOLIO

The objectives of SCORE were to 1) evaluate alternative approaches to gaining and sustaining control of schistosomiasis and to eliminating schistosomiasis where possible, 2) develop mapping and diagnostic tools needed for a global effort to control and eliminate schistosomiasis, and 3) assist in moving SCORE findings into practice. The SCORE portfolio was broad—from large field intervention studies, to laboratory- and field-based assessments of diagnostic tests, to assessing snail abundance and shedding as a measure of force of transmission, to schistosome population genomic studies (Table 2).

**Table 2:** Major studies in the SCORE portfolio

Study name	Study type	Study description
Gaining and sustaining control studies	Cluster-randomized trials	Villages were randomized to receive various MDA regimens over 4 years. Major outcomes were prevalence and intensity in schoolchildren at the end of the study in the fifth year.
Cohort morbidity studies	Prospective cohort studies nested in gaining control studies	Children were evaluated at baseline, Year 3, and Year 5 for multiple potential morbidity markers.
Niger once- vs. twice-a-year MDA	Cluster-randomized trial	After 2 years as separate gaining and sustaining control studies, the Niger studies were combined and redesigned. Villages from both studies were randomized to test the benefits of once- vs. twice-a-year MDA on prevalence and intensity.
Studies of predictive factors for PHS in Kenya and Tanzania	Surveys, focus groups, and key informant interviews	Evaluations of potential factors contributing to a village being a PHS vs. a responder village were conducted in villages meeting these criteria following 4 years of MDA in the gaining control studies in Kenya and Tanzania.
Zanzibar elimination study	Cluster-randomized trial	Shehias were randomized to one of three study arms over 5 years: biannual MDA, biannual MDA + snail control, biannual MDA + behavioral change intervention. Major outcomes were prevalence and intensity in schoolchildren at the end of the study in the sixth year.
Seasonal elimination study	Cluster-randomized trial	Villages were randomized to one of four study arms: annual MDA before peak transmission season, annual MDA after peak transmission season, twice-a-year treatment before and after the peak transmission season, and MDA + snail control before peak transmission season.
POC-CCA assessments, including the five-country study and mapping in Burundi and Rwanda	Laboratory and field assessments	Laboratory studies assessed aspects such as batch variability and inter-user reading variability.
		Field studies compared POC-CCA results to those from Kato–Katz and assessed implications of egg-negative CCA-positive test results.
Up-converting lateral flow phosphor circulating anodic antigen improvements and assessment	Laboratory studies to increase sensitivity and use to validate field results	Laboratory research was carried out to improve test performance and sensitivity to detect one worm pair in experimental infections in baboons.
		Specimens from Zanzibar, Burundi, Rwanda, Tanzania, and St. Lucia were tested to complement results from other measures of prevalence and intensity.
Snail prevalence studies to assess force of transmission	Field and laboratory studies, including speciation and assessment of pre-patency	Prevalence of snails and infected snails at transmission sites was evaluated as a potential measure of force of transmission as part of Côte d’Ivoire’s seasonal study and field studies in, Niger, Tanzania, and Zanzibar. Other studies included an ecological study of prawns, snails, and human infection in Côte d’Ivoire; a xeno-monitoring study in Tanzania; and a study of snails in villages that were identified as PHS and villages identified as responding to MDA in Kenya.
Schistosome population genetics studies	Laboratory analysis of genomic patterns of schistosome and human/livestock schistosome hybrids	Specimens were collected from snails and children in Niger, Tanzania, and Zanzibar, and the Côte d’Ivoire seasonal elimination studies to assess potential development of resistance in response to drug pressure and, in some cases, female worm fecundity related to adult worm density.
RAP	Systematic reviews of existing data	Seven RAPs were conducted to evaluate questions arising during the development and conduct of the SCORE studies.
SCORE modelling studies	Mathematical modelling studies	SCORE and other data have been used to explore the impacts of alternative approaches to control and elimination in a range of transmission settings.

MDA = mass drug administration; PHS = persistent hot spots; POC-CCA = point-of-care circulating cathodic antigen; RAP = Rapid Answers Project; SCORE = Schistosomiasis Consortium for Operational Research and Evaluation.

Although managing such a broad range of activities is challenging, the benefits of having a diversified portfolio are manifold. For example, by the Year 3 parasitological survey in the large field studies of gaining and sustaining control,^2^ it became clear that around 30% of villages studied were persistent hot spots (PHS)—villages that failed to decline in infection prevalence and/or intensity despite several rounds of adequate mass drug administration (MDA).^3^ To answer questions about why some villages were PHS, SCORE built on the relationships and infrastructure that had been established within the consortium to quickly implement additional studies, some of which linked SCORE researchers from the gaining control studies with those working on snails and parasite population genetics. In another example, SCORE support for Leiden University Medical College to improve the up-converting lateral flow phosphor circulating anodic antigen (UCP-LF CAA) test contributed to SCORE assessments of point-of-care circulating cathodic antigen (POC-CCA) performance in low-prevalence *Schistosoma mansoni–*endemic areas and also helped better define the true prevalence of schistosomiasis in several areas within ongoing SCORE field studies.^4,5^

## BROAD ENGAGEMENT BY THE SCHISTOSOMIASIS COMMUNITY

SCORE’s origins lie in an early effort by Dan Colley and Evan Secor to develop a community-informed schistosomiasis research agenda. Ideas were solicited from 350 people, and 150 people contributed.^6^ The operational research aspects of this agenda were the basis for the initial BMGF interest in supporting SCORE.^1^

The commitment to obtain broad input continued as SCORE’s research studies were being defined. For example, in the first year of funding, SCORE held seven expert panels involving people whose primary focus was schistosomiasis and others having relevant complementary and critical expertise. Although there were financial costs and time investment associated with these meetings, these and subsequent meetings provided SCORE with a wide range of ideas and experience and helped SCORE develop relationships and linkages to support rapid dissemination of SCORE results throughout the research and practice communities.

### Partnerships with country-neglected tropical disease (NTD) control programs.

Operational research questions can often be answered efficiently through collaborations by academic institutions with affiliated sites. Although this approach often produces quality results and publications, it may not be optimal for fostering integration of findings into policies and programs.^7^ Therefore, SCORE required partnership with NTD control program leadership in all major field studies.^1^

Collaboration and engagement between investigators and programs was not always easy. In some places, there was rapid turnover of program managers; in others, there was a history of discord; and in others, there had been little prior relationship. For example, before SCORE, in Côte d’Ivoire, the national NTD control program had little interaction with a long-standing active schistosomiasis research program housed at Université Félix Houphouët-Boignya in Abidjan, which has been the in-country principal investigator (PI) for two large SCORE field studies. Following a meeting in Côte d’Ivoire and participation of the Côte d’Ivoire NTD control program director in SCORE annual meetings, relationships were established between research and program that we expect will continue.

### Participation of the World Health Organization (WHO).

The WHO guidelines for schistosomiasis at the time SCORE was started were based on limited information, mainly related to severe morbidity.^8^ Their appropriateness for the evolving context, in which prevalence and intensity had been lowered because of extensive preventive chemotherapy (PC), was unclear. Because SCORE studies were designed to contribute to an evidence base for WHO guidelines, the involvement of the WHO headquarters’ NTD leadership in planning SCORE studies and as an active ad hoc member of the SCORE Advisory Committee has been essential, and SCORE findings are now contributing to WHO guidelines related to the use of the POC-CCA urine test for detecting *S. mansoni* infection and MDA in the control and elimination of schistosomiasis.^9^

## TRADE-OFFS AND BALANCES

Many aspects of SCORE involved trade-offs and balancing of competing priorities. Examples of hard decisions included how to allocate the finite SCORE resources; how much was reasonable to ask of research institutions, field sites, and NTD control programs, particularly for the large field studies; and where to conduct studies. Another important issue in the large field studies was how to balance the need for rigorous, carefully controlled field studies with a desire to conduct studies in contexts similar to what program managers would encounter, where many aspects of program implementation and data collection can be challenging.^7^

### The ideal versus resources and capacity.

The large investment in SCORE by the BMGF created great excitement in the schistosomiasis research community. Not surprisingly, the ideas about possible research opportunities far exceeded the available resources. For example, multiple options were proposed for new screening tests, in particular to move from dependence on stool examination for *S*. *mansoni*; for true diagnostics for all *Schistosoma* species; and for field studies related to control and elimination of schistosomiasis.

At the time SCORE started, supplies of praziquantel were limited and costly. Although the research and practice communities were heartened by funding of SCORE to conduct large trials related to schistosomiasis control, there was also disappointment that it was clear that many other important questions and issues could not be addressed. Examples of good, important questions related to gaining and sustaining control that were not initially included in SCORE’s portfolio were effectiveness of twice-a-year PC by MDA, results of various multiyear MDA regimens when MDA with praziquantel was included as part of an integrated NTD control program versus as a stand-alone treatment, and how best to treat sites endemic for both *S. mansoni* and *Schistosoma haematobium*.

With broad input, the SCORE secretariat narrowed the field research questions about schistosomiasis control to what became the gaining and sustaining control studies.^10^ Decisions about the study design, number of regimens to be evaluated, and number of individuals to be recruited were impacted by practical considerations, including the programmatic and financial resources available. The six-arm gaining control studies^2^ in particular involved a massive amount of field and laboratory work; adding more arms to test more regimens was not feasible. For example, in Year 1, each of the gaining control studies required sampling up to 37,500 individuals and, for studies in *S. mansoni* areas, preparing and reading more than 135,000 Kato–Katz stool slides. Although consideration was given to increasing study power by increasing the number of villages from 25 per arm, the logistics of enrolling more than 150 villages, even if the number of individuals per village was decreased, were daunting. It is a tribute to the dedication of investigators and field teams that these studies have been completed and have contributed substantially to the knowledge base about control of schistosomiasis.

### Balancing rigor and relevance in the SCORE field studies.

The large field studies yielded many critical findings. For example, praziquantel MDA was effective in reducing average *Schistosoma* prevalence and intensity of infection in all arms of all studies. Importantly, for WHO guidelines, even before MDA initiation, many villages in areas with moderate to high prevalence of schistosomiasis met the WHO criteria for control of morbidity and elimination as a public health problem,^11^ suggesting that these need to be redefined. The finding of around 30% PHS has led to several follow-up efforts to understand how to identify these earlier in a multiyear MDA effort and how to respond to them. Some of the challenges and compromises that went into designing and completing these studies are described in the following text.

#### Issues related to the cluster-randomized design in the large field studies.

A cluster-randomized design was chosen for the large field studies of gaining and sustaining control and the Zanzibar and seasonal elimination studies because the interventions were delivered at the village level and were expected to impact not just the individuals who received the interventions but also others who could be protected or put at risk by behaviors of others in the community.^12^ However, when an intervention is assigned randomly to a group, potential confounders may not be balanced among groups; at least in some SCORE studies, it appears that study arms were not balanced. In future studies, conducting a baseline survey followed by a stratified randomization could be considered. Another issue that reduced the ability to detect significant differences between arms is that variability in village-level responses to MDA was much greater than anticipated, largely due to PHS.^3,12^

Because our primary outcome was infection prevalence and intensity in 9- to 12-year-old children, we used schools as the site for recruiting children for parasitological evaluation. We assumed that children attending a given school would be exposed to the intervention applied to the village where the school was located. Evidence suggests that may not have always been the case. In Kenya’s gaining control study, up to 10% of children in Year 1 identified their home village with a name that was different from the name of the village where the school was located, although not all of these may have represented truly different villages.

Another issue was that children may have been exposed to interventions occurring in villages other than those they lived in through nonschool activities. In Zanzibar, although an effort was made to ensure that snail control shehias (the lowest official administrative unit in Zanzibar and the unit used for randomization) did not share water sources with those that did not have snail control, children in study arms without snail control may have visited streams or other water sources that were in snail control areas, in addition to water sources near their homes.

Randomization also created issues for those distributing MDA because NTD control programs are typically implemented using administrative boundaries. Having nearby villages in a single administrative unit receiving different treatments led to both logistical and communication challenges.

Future studies may need to pay more attention to ensuring that the potential for “contamination” of arms is minimized, by ensuring that individuals in one arm are not exposed to interventions in another arm. More care should also be taken to ensure that the populations receiving intervention and those being tested to measure impact are the same. Attention to community drug distributor (CDD) training and to communication to villages about why differential treatment is occurring is also crucial.

#### Working in the context of ongoing programs.

For the gaining and sustaining control and elimination studies, a critical tension existed between the desire for studies that would be rigorous versus ensuring relevance and immediate applicability of study results. Conducting the research in the context of ongoing programs resulted in some aspects of the studies, including MDA with praziquantel—a critical intervention, and sometimes the only intervention, in the field studies—not always being conducted optimally. For example, some communities did not receive adequate sensitization before MDA, and some villages received different treatments than per protocol. In November 2014, in the Zanzibar elimination study, the Ministry of Education did not permit the sixth round of MDA to be conducted in schools in Unguja because of the school examination period.^13^ In the Tanzania gaining control study and the Zanzibar elimination study, pregnant women were not treated, contrary to the agreed-to protocol, but consistent with country policy and practice. Other issues related to coverage are discussed in the following text and in detail in the article on coverage.^14^

Another issue was the balance between specifying interventions and measurements in great detail in multisite studies versus providing flexibility for local context. In hindsight, more aspects of the processes around MDA and coverage measurement should have been specified. For example, we chose not to dictate MDA processes that we believed were routine and might differ among study sites, such as community sensitization. Details of CDD training and supervision were also left to individual studies. In some places, this worked better than in others. More timely and consistent collection and evaluation of data on process measures might have allowed for earlier recognition of issues.

Given that SCORE provided resources for additional sensitization and other implementation activities, it is possible that the results from MDA in these studies represent the best that can be expected from routine MDA programs. However, because nearby villages were randomized to different MDA schedules, the study design might have made MDA delivery more difficult than when it is provided to all villages in a large area.

### Choosing study sites.

#### Ensuring findings would be generalizable: The Zanzibar elimination study.

SCORE’s initial grant included funding for a major study on elimination of schistosomiasis. After an extensive assessment of a range of possible locations for the study,^12^ the decision was made to work in Zanzibar. Zanzibar is a clearly defined geographic area with strong political commitment and a long history of an effective praziquantel MDA program. A major concern about conducting a cluster-randomized study in Zanzibar was the limited number of schistosomiasis-endemic shehias. Because some shehias were near each other or potentially shared water sources, individuals from one arm could be exposed to interventions from another. There were also concerns that findings for island ecosystems would not be perceived as generalizable to the mainland.

The study in Zanzibar demonstrated that prevalence and intensity can be reduced even in areas that have achieved the WHO criteria for elimination as a public health problem, but that interruption of transmission will be difficult. Generalizability of the findings to the mainland is no longer being questioned, and results are being widely cited.^13,15–17^ However, the limited number of shehias for evaluation and their proximity to each other are among factors that may have contributed to an inability to find significant differences between arms.^12,13^

#### Balancing risk and opportunity in site selection.

In several of our studies, research sites of interest were in areas that were thought to be politically unstable or where there were security risks. The decisions to conduct studies in these settings involved careful weighing of the potential knowledge to be gained at these sites with that to be gained among the alternatives, while recognizing that potentially unstable situations can change quickly (and that stable sites can become unstable).

In the gaining and sustaining control studies, some of the sites with the most expertise in MDA and research related to schistosomiasis and strong track records were in areas emerging from conflict, such as Côte d’Ivoire, or deemed unsafe for travel, such as Niger. There were concerns about the field site in Cabo Delgado Province, northern Mozambique, because it had almost no experienced researchers and limited infrastructure to conduct MDA and collect data. It is remote, making oversight difficult. However, the lack of prior MDA and very high levels of *S. haematobium* infection made it attractive as a site for a gaining control study.

In 2013, SCORE received supplemental funding for additional research on approaches to achieving elimination. With the support of the national governments, SCORE project feasibility assessments were implemented in Rwanda and Burundi, two countries that had been working with the Schistosomiasis Control Initiative (SCI) and the END Fund to substantially reduce schistosomiasis prevalence. From the start, there were concerns about the political and security situation in Burundi and whether extensive intervention research would have the support of the government of Rwanda. Nevertheless, the opportunity to compare the impact of combined interventions aimed at eliminating schistosomiasis in areas that were ecologically similar but with important differences in governments, economies, etc., was attractive.

In 2014, extensive mapping in Burundi and Rwanda with Kato–Katz and POC-CCA assays and testing of a subset of specimens with the UCP-LF CAA assay showed that there was much more schistosomiasis than had been thought.^4,5^ The next planned step was to develop intervention studies in the two countries. In Rwanda, government backing for the study was not obtained. In Burundi, a constitutional crisis occurred before the study could be designed and implemented, making it impossible to move forward. Although the large intervention trials did not materialize, the data collected related to POC-CCA performance have been invaluable in assessing the assay’s performance in low-prevalence areas.^4,5,18–20^

### Using new technologies.

Studies that span many years may be accompanied by technologic developments that can make the work easier and cheaper. Striking the right balance between embracing new technologies and using proven, currently practiced, but potentially less efficient methods can be challenging.

#### Data collection.

In early consultations about the gaining and sustaining control studies, leaders from the NTD community and PIs encouraged SCORE to provide personal digital assistants (PDAs) to help with data collection. By the time the studies were nearly ready to go into the field, the use of PDAs was phasing out worldwide in favor of smartphones. After assessing several options, the decision was made to build on existing software for use on Android phones that had been developed by the EpiCollect team based at Imperial College for use in ecologic studies.

SCORE was under pressure to get into the field quickly. The first studies in the field, in Kenya, began before data collection software had been fully developed and tested. Most of the major problems we encountered in establishing data collection systems could have been avoided had there been more time for development before initiating fieldwork or had our studies begun a few years later, once vetted phone-based data collection software was more widely available. Also, in retrospect, the system we envisioned was comprehensive and, therefore, very complex. The software development team probably had insufficient experience and personnel to guide us to focus on a simpler design with less elements and linkages and to focus sufficiently on the end-user experience. Those developing the software did not anticipate critical logistical issues related to data cleaning and security that in retrospect appear obvious, such as including checks to ensure that each individual in a study was given a unique identification number.

Because of delays in obtaining human subjects’ approval and receiving praziquantel, the study in Tanzania started almost a year after Kenya’s. Data collection in Tanzania benefited from the experience in Kenya and went somewhat more smoothly. Because of its civil war, Côte d’Ivoire started its study a full year after Kenya began. By the time Côte d’Ivoire was ready to collect data, Open Data Kit tools for designing data collection software had markedly improved and became the basis for their data collection system. Despite difficulties, in-country staff from all studies were enthusiastic about being at the cutting edge with technology and persevered through issues related to the phones, software, and internet access.

It is easy to call for using proven technologies and software for data collection and for designing and testing systems in advance. However, as long as tools for data collection continue to rapidly improve and the types of data and information that can be easily captured expand, large, multiyear projects will continue to be challenged to use technologies that are new enough to be acceptable several years into the project although not being so new as to make the project unworkable.

#### Genomic analyses.

As part of the studies of gaining and sustaining control and the Zanzibar elimination studies, substudies were designed to evaluate the effect of MDA on the population structure of parasites, both from humans and from snails. Questions included whether schistosome population data could be used as a measure of control outcomes and success and whether genetic bottlenecking, if seen, could be an early bellwether of the development of drug resistance. When PHS were identified as a significant issue, the question of whether the schistosome population structure was related to a village’s response status was also assessed.

SCORE recognized that the costs of DNA sequencing were likely to fall dramatically over time. Therefore, the emphasis in the first 3 years of the schistosome genomic work was on collecting specimens and developing more cost-effective assay systems, with most genomic sequencing and complex analyses deferred until later years. Even in later years, the assessments were limited to those most likely to help understand whether intensive MDA was increasing the risk of developing resistance.^21^

The amount of genomic work accomplished with the available resources was far more than would have been possible had extensive investment been made in genomic analysis early in the study instead of waiting for development of new processing techniques and technologies to lower costs.^21^ The results to date do not indicate decreases in diversity of schistosomes or other early indicators that resistance was being fostered, even in SCORE study sites with the most intensive MDAs. However, the results have been intriguing. For example, hybrids of *S. haematobium*–*Schistosomaa bovis* occur with frequency at some sites, suggesting the need for further research related to morbidity from these and their contribution to ongoing transmission among humans.^21^

Because of SCORE’s decision to wait for technologic developments before supporting extensive genomic analysis, many important questions are not yet answered. All SCORE parasite samples are archived in the Schistosomiasis Collection at the Natural History Museum (https://www.nhm.ac.uk/our-science/our-work/sustainability/schistosomiasis-collection.html). With the increased availability of genome sequences and recent advances in DNA sequencing technologies, future evaluations will likely produce further understanding of the impact of treatment on parasite genomics.

## MANAGEMENT AND OVERSIGHT

### The SCORE secretariat.

To provide as much resources as possible for research and to encourage country and PI ownership, the SCORE secretariat was purposefully kept to a small core team. We benefited from the support of an active multidisciplinary Advisory Committee and an in-house affiliation with the UGA College of Public Health. However, we chose not to allocate resources for additional staff and extensive travel needed for more continuous, ongoing in-country oversight, partly based on the established links between endemic countries and their northern partners.^1^

Approaches to oversight of all studies involved frequent phone and email contact, written annual reporting, and presentations at SCORE annual meetings, which brought together all SCORE investigators. Additional meetings were scheduled during conferences attended by the secretariat and SCORE researchers, such as meetings of the *American Society of Tropical Medicine and Hygiene*. Site visits were also made to all field studies where conditions permitted (see in the following text) and to research partner institutions.

In retrospect, the amount of oversight provided was not sufficient, as described in specific examples in the following text. There was no site that implemented the entire protocol exactly as agreed to and as articulated in the final, signed protocols. Future studies should include resources for careful oversight, with site visits occurring before and during start-up and more regularly thereafter.

### Leadership of individual studies and secretariat oversight.

All the funded gaining and sustaining control studies included at least one PI with a track record as a senior scientist working on schistosomiasis from Europe or the United States (the northern partners) and a PI from the African country where the study would take place. Based on input from planning meetings, the expectation was that this would be sufficient to ensure full compliance with the agreed-to protocol and signed sub-award agreements.

During start-up, SCORE’s associate director for management, visited Kenya, Tanzania, and Mozambique. Travel to Côte d’Ivoire and Niger was not possible for security reasons. Among the problems he identified were that Mozambique was reading one urine filtration instead of two, and some villages in Tanzania, which had been identified by the investigators as having only *S. mansoni* infections, were found to have prevalent *S. haematobium* infections as well*.*

Additional problems with performance became obvious at the first annual meeting of SCORE investigators. The initial problems were largely attributed to start-up, and specific plans were made to address these as individual and not systematic problems. Subsequently, additional major problems were identified, such as failure to use schools as one of the MDA venues in villages assigned to community-wide treatment and lack of strong social mobilization before MDA, with resultant low coverage among school-aged children.

After the second year of each large field study, SCORE secretariat members and a representative from the SCORE Advisory Committee made site visits to the large projects and conducted more in-depth assessments of fieldwork and the data being collected. The mid-study review of the Niger program was held in Côte d’Ivoire because security issues continued to preclude secretariat travel to Niger. Multiple protocol violations were identified, the most serious being the failure in Niger to randomize villages appropriately; as a result, the Niger study had to be restructured, and SCORE was left with no study of sustaining control and only one study of gaining control (Mozambique) in areas with *S. haematobium*.

### Resources for contingencies and opportunities.

It was recognized by all engaged in SCORE—including the BMGF, the secretariat, and the PIs—that the sub-awards to all investigators were only guesses as to what funds and other resources might be required to conduct the studies. Supplemental funding was needed for a range of issues outside of the investigators’ control. For example, delays in obtaining samples due to school closures during elections required laboratory staff to be retained for additional months. We are grateful to the BMGF for the flexibility provided to us and pleased with the results of the add-on efforts that we supported.

The SCORE budget suffered a potentially major setback early on, when the WHO determined they could not deliver on their promise to supply praziquantel for the SCORE gaining and sustaining control studies. Graciously, SCI, the U.S. Agency of International Development, and the U.K. Department for International Development agreed to provide most of the needed drug. In other countries, through SCORE’s intercession, NTD control program managers provided expiring drugs to programs in nearby countries. SCORE’s net drug purchases for these studies were well more than $100,000 but would have been much higher if not for the much-appreciated support of many other organizations and individuals.

SCORE’s ability to support add-on studies as questions arose yielded critical data. For example, Kenya conducted studies related to reading of POC-CCA tests and changes in POC-CCA results in children initially testing positive after they were treated,^22^ and Kenya and Tanzania conducted studies of PHS identified during the gaining control studies.^3^ Other relatively small studies were designed to answer questions arising from the large studies or from diagnostic development work. For example, SCORE supported research in Egypt to assess whether children who were POC-CCA positive, in areas that *S. mansoni* transmission was believed to be minimal or nil, were actually excreting eggs.^23^ In that study, only one stool of 1,388 consecutive specimens tested contained an egg.

## INTERVENTION IMPLEMENTATION ISSUES

### Gaining and sustaining control studies MDA implementation.

As part of the gaining and sustaining control studies, an estimated 3.5 million treatments were provided—an impressive number. However, the total and the average coverage across studies^14^ mask very high and very low coverage rates in some places. Perhaps because MDA was seen as a programmatic issue, much of the focus of the PIs and the SCORE secretariat was on ensuring the quality of the parasitological data, particularly in the gaining and sustaining control studies. Given that MDA was the primary intervention, in retrospect, the SCORE secretariat and the researchers should have invested more resources and attention into achieving and measuring coverage.

Many of the problems encountered in attempting to achieve high coverage were outside the studies’ control. These included political instability, teacher strikes, floods, severe food shortages, and cholera and other disease outbreaks. However, some of these could have been foreseen and plans made to address them or at least record them consistently and systematically across studies. Some issues that affected coverage could potentially have been resolved by engaging more directly with policy leaders and CDDs, for example, the failure to include pregnant women in MDAs in Tanzania and Zanzibar.

Provision of food during MDAs was discussed several times during the design of the studies and at SCORE annual meetings. Ideally, food would have been included in all praziquantel MDA campaigns to reduce side effects, encourage participation, and potentially enhance absorption of praziquantel.^24,25^ For the gaining and sustaining studies, it was decided that SCORE would not pay for food, both to preserve resources and to emulate what the national programs reportedly were doing.

We also did not plan on how to prepare for the impact of food shortages. In Kenya, from the perspective of the CDDs, concerns about food were a contributor to MDA noncompliance.^26^ Therefore, in Kenya, CDDs adjusted their home visit times to when people were likely to have eaten. In the study area in Mozambique, the estimated cost of juice and biscuits provided in an MDA after the SCORE study was completed was less than $0.10 per person treated, suggesting that providing food need not be as expensive as had been anticipated and perhaps should have been included in all studies, regardless of programmatic concerns.

Other issues that impacted both coverage and measurement of coverage could have been anticipated. For example, all field study MDAs and parasitological surveys involved village schools, so when school catchment areas changed or new schools opened in study areas, adjustments had to be made. Such issues should have been recorded in the annual village inventories collected by the study teams (see in the following text), but they were not always captured. In retrospect, SCORE could have invested more in higher quality data collection related to village-level variables that could have impacted results and conducted more timely and ongoing analysis to identify events that were impacting multiple studies (and suggest responses) and modify data collection tools if needed.

Problems related to measuring coverage are described in detail in another article in this supplement.^14^ Stricter rules related to what coverage data would be considered acceptable and better investigation of reasons for out-of-range coverage levels (reported village-level coverage was 694% in one village) might have improved implementation and measurement of this critical aspect of MDA intervention.

At the time SCORE studies were being developed, there was no consensus on how best to conduct coverage surveys, so they were not required in SCORE studies if quality numerator and denominator data could be obtained. Since then, the WHO has published evidence-informed guidance and detailed methodologies. A recommendation from our experiences in these field studies is that if MDA is a primary intervention, study protocols should strongly consider including formal coverage surveys using recommended approaches.^27,28^

### Zanzibar elimination study implementation.

The Zanzibar elimination study MDA encountered some of the issues seen in the gaining and sustaining control studies, such as political issues or other concerns delaying MDAs or resulting in MDA not using schools as venues.^29^ Delays in money transfers between SCI and the Zanzibar Ministry of Health sometimes resulted in delays in MDA implementation. The other major interventions in this study—behavioral change and snail control—also provided challenges.

Regarding snail control, challenges included identifying all the relevant water bodies and assessing the large number of natural freshwater bodies, especially because many of them were remote and some were seasonal. Niclosamide was only applied to human–water contact sites where infected snails were found to minimize ecologic impacts and conserve resources, so it is likely that some areas with transmitting snails were not treated. Even in treated areas, because snails are hermaphroditic, even a single snail could have quickly repopulated a treated area. Currently, the implementation of snail control is limited in Africa. It is hoped that future studies involving snail control will take advantage of the expertise developed during the SCORE studies. Further research will be needed on the best ways to identify human–water contact sites with infected snails and optimization of focal niclosamide application and other approaches to snail control.^30^

In Zanzibar, the behavioral change component used a human-centered design approach.^31^ Although the community engagement was a critical part of the intervention, specific aspects of the behavioral change program as designed were not implemented until the third year of the study. Even then, they were implemented incrementally and not as fully as desired because of resource limitations. Clearly defining behavioral and other interventions in advance allows for faster implementation but potentially at the cost of community buy-in and being a good match to the local context.

## MEASUREMENT AND ANALYSIS OF OUTCOMES

### Standardization of data elements and explicit data cleaning processes.

An early assumption of the SCORE secretariat was that the seasoned researchers conducting gaining and sustaining control studies would ensure data submitted to the secretariat was complete and clean. However, the early data included problems such as multiple individuals with the same person ID number, village names that differed from those that had been randomized, child ages that were much higher or lower than the SCORE study cutoffs, and missing data.

At first, data checking was conducted at UGA, with queries sent to investigators when problems were found. However, this resulted in multiple back-and-forth volleys and was extremely time-consuming. In 2014, the SCORE secretariat established specific rules regarding what data would be accepted, for example, each individual had to have a unique identifier and ages had to meet study limits. After checking by the SCORE secretariat, any dataset with errors was returned to the PIs in its entirety. SCORE also developed and established the SCORE uniform data set (SUDS) requirements, which included a data dictionary with specific formats for data and requirements for reporting of data problems. A great deal of unnecessary work might have been avoided had the SUDS and required data checks been defined before sites beginning data collection. With a few exceptions, all data from SCORE’s major field studies are now in SUDS. Starting in 2020, anonymized individual participant data and accompanying data dictionaries for the field studies will be housed and made accessible through Clinical Epidemiology Database Resources (https://clinepidb.org/ce/app/) at the University of Pennsylvania.

### Standardized analysis plans.

The protocols for the large field intervention studies specified the outcomes of interest, for example, comparison, by arm, of changes in prevalence and intensity among 9- to 12-year-old schoolchildren in the gaining and sustaining control and Zanzibar elimination studies, and a variety of biometric and other health indicators in the morbidity cohort studies.^10,32^ In addition, a UGA statistician developed more rigorous plans, for example, describing the specific statistical tests that would be used to compare performance of arms and addressing issues related to multiple comparisons.

In 2017, the BMGF requested that SCORE formalize the study hypothesis-based standardized analysis plans (SAPs) for all the large field studies at that time—the gaining and sustaining control, morbidity cohort, Niger, and Zanzibar elimination studies. This effort engaged statisticians and investigators from the secretariat and all study sites. The resultant documents were designed to provide a uniform roadmap for presentation and analysis of the data, including flowcharts describing recruitment and loss to follow-up; specifications for presenting descriptive analyses; statistical models to be used; and, for studies involving repeated cross sections, designs for graphs showing village-level variability in response to MDA.

For randomized controlled trials, the modern standard is to prepare a SAP closely tied to study design, before beginning the study, to ensure that the protocol has been followed and that reporting of results is not biased by selective analysis to support post hoc hypotheses. The SCORE SAPs were peer-reviewed and finalized after protocol development and during the ongoing implementation of most studies, but before final data were available and final study outcomes analyzed. However, the experiences and issues identified during the first years of the study, for example, related to the randomization, helped ensure that the SAPs addressed issues that had not been anticipated at the outset of the studies. The SAPS are available as supplementary files to other manuscripts in this journal supplement.^2,13^

### Measurements intended for additional analyses.

#### Village-level data.

Among the issues of interest in the gaining and sustaining control studies was whether simple measures potentially related to force of transmission would contribute to our understanding of the results. Therefore, a village inventory was performed annually by the study teams, usually by interviewing village leaders and other key informants. It addressed issues such as water and sanitation, types of work conducted in the area, proximity of health facilities and stocking with praziquantel, and types of nearby water bodies. After the first year, the inventory asked about environmental events such as floods or droughts, major shifts in population or occupations, and administrative changes.

Unfortunately, these village inventories did not prove useful. One problem was that the forms and data collection processes had not been field tested before use. Possibly because different people in the villages provided information in different years and data were not independently verified, estimates varied greatly across years. Inclusion of village inventory data did not improve performance of mathematical models comparing parasitologic outcomes in different villages. Subsequently, more rigorous studies were conducted comparing village-level data in PHS with data from responder villages in Kenya and Tanzania.^3^ Results of these are pending. Future studies may want to either invest in obtaining quality village-level data or forgo the types of village-level collection included in the SCORE gaining and sustaining control studies.

#### Cost evaluations.

Besides evaluating the impact of different MDA regimens in the gaining and sustaining control studies, SCORE tried to determine which would be most cost-effective to achieve a given change in prevalence. The SCORE cost evaluation was developed with the help of individuals who had conducted studies related to cost-effectiveness of MDA for other NTDs in the past. The forms were simplified versions of the forms that had been used in past studies. Special instructions were developed to help sites distinguish research costs (which were not of interest in this analysis) from program costs.

Training was conducted for the PIs from all sites during the SCORE annual meetings in 2013 and 2014. Principal investigators were to then train their staff and oversee cost data collection. SCORE secretariat staff and the economists involved in developing the tools were available to answer questions. The data received from most of the sites were difficult to clean, and analyses yielded results that were inconsistent or uninterpretable. The only study that fully implemented on-site training was the Kenya gaining control study; however, because so many of their costs were related to the use of vehicles and other assets from the CDC facilities in western Kenya, the costs were not generalizable to a typical program and were not published. However, the Kenya team broadened the scope of the evaluation to compare costs of using single stool Kato–Katz, triplicate stool Kato–Katz, and POC-CCA for mapping, which provided useful data.^33^ In retrospect, SCORE should have more directly implemented and supported on-site training for those who would be conducting cost data collection and oversight to ensure compliance with the protocol and uniform data entry.

## CAPACITY BUILDING

Under the BMGF grant to SCORE, capacity building was only allowable if essential for the implementation of SCORE projects. Nevertheless, the consortium was creative in building capacity wherever possible. Obviously, conducting the range and scope of the SCORE studies required training of field-workers, laboratorians, data managers, information technology specialists, and those with several other skills. Laboratories and information technology infrastructure were upgraded as needed. The use of smartphones for data collection in the gaining and sustaining control studies, as mentioned, was a wonderful experience for field-workers in several countries. Several projects engaged junior researchers, providing them with new skills and opportunities. The teams in Kenya and Côte d’Ivoire were particularly creative in finding opportunities for new public health professionals to participate in study design, data collection and analysis, writing of articles, and use of data for advanced degree work. The inclusion of snail control in several SCORE studies had a large impact on the field.^30^ Even if explicit resources are not provided to build human resource capacity and infrastructure, studies can work creatively to maximize opportunities to build human capacity and infrastructure.

## FINAL WORDS

The danger in writing a “lessons learned” article is that the negative experiences receive more attention than the positive ones. In a way, running and participating in a large research consortium is similar to public health—when it works well, the hard work and successes get little notice. An article summarizing lessons learned by its nature includes many stories of things that did not go right and can leave an inappropriate and false impression of a suboptimal project and results. The bottom line is that the SCORE consortium conducted an enormous amount of work that is already benefiting communities endemic for schistosomiasis and that will have a lasting impact on future efforts to control and eliminate this disease. The SCORE secretariat and the schistosomiasis community learned many valuable lessons in carrying out SCORE. We hope that others can benefit from our positive and negative experiences and can see the issues captured in this article as part of the overall outcomes from SCORE.
